# Deep Vein Thrombosis Prophylaxis in Orthopedic Surgery

**DOI:** 10.7759/cureus.53726

**Published:** 2024-02-06

**Authors:** Shu Lin, Adrian Alepuz, Tara Tritsch, Gary Schwartz

**Affiliations:** 1 Medical Education, Nova Southeastern University Dr. Kiran C. Patel College of Allopathic Medicine, Fort Lauderdale, USA; 2 Orthopedic Surgery, Nova Southeastern University Dr. Kiran C. Patel College of Allopathic Medicine, Fort Lauderdale, USA

**Keywords:** dvt prophylaxis, orthopedic surgery, postoperative complication, deep vein thrombosis (dvt), cost-effectiveness

## Abstract

Deep vein thrombosis (DVT) is a complex and multifactorial process arising from a variety of factors, including recent surgical procedures, traumatic events, and periods of prolonged immobility. The extended period of stasis post-orthopedic surgery places patients at a notably high risk of developing DVT, and DVT-related pulmonary embolism (PE) ranks as the third most common cause of death in orthopedic surgery patients. This review examines the multifaceted risk factors contributing to the development of DVT in orthopedic patients. Additionally, it addresses the importance of DVT prophylaxis in orthopedic settings, the efficacy and safety of various prophylactic methods encompassing both mechanical and pharmacological approaches, and the economic dimensions of DVT prophylaxis, including scrutiny of cost-effectiveness and the exploration of strategies for optimization.

## Introduction and background

Deep vein thrombosis (DVT) is a life-threatening medical condition characterized by the formation of blood clots within deep veins, typically occurring in the lower extremities or pelvis. In the United States, it is estimated that over 900,000 individuals are susceptible to DVT annually, resulting in a significant public health concern [1]. Moreover, DVT is associated with a substantial mortality rate, with 60,000 to 100,000 reported deaths attributed to this condition each year. Approximately 33% of individuals who experience DVT subsequently endure long-term complications, including limb swelling, pain, discoloration, and skin scaling [1]. Virchow's triad, consisting of endothelial damage, stasis of blood flow, and a hypercoagulable state, has traditionally been recognized as the primary factor predisposing individuals to DVT development [2]. These factors collectively increase the risk of venous thrombosis, manifesting with symptoms such as limb swelling, pain, and tenderness. Of note is the variable incidence of DVT across different populations. Orthopedic surgical procedures, especially those involving the lower extremities, such as total hip or knee arthroplasty, present a heightened risk of DVT development. This elevated risk is primarily attributed to the prolonged postoperative immobilization of patients, which can lead to stasis and significantly increase the likelihood of DVT occurrence, affecting up to 60% of patients [3].

Deep vein thrombosis is a complex and multifactorial process arising from a variety of factors, including recent surgical procedures, traumatic events, and periods of prolonged immobility [4,5]. Diagnosing DVT can present challenges, as many affected individuals may either remain asymptomatic or exhibit nonspecific symptoms [5,6]. This underscores the importance of conducting appropriate diagnostic tests promptly to confirm or rule out DVT. Early intervention in cases of DVT is of paramount importance to mitigate the potential for severe complications. Among these complications, post-thrombotic syndrome and recurrent venous thromboembolism (VTE) stand out as significant concerns [7].

The extended period of stasis post-orthopedic surgery places patients at a notably elevated risk of developing DVT [8-11]. In fact, DVT-related pulmonary embolism (PE) ranks as the third most common cause of death in orthopedic surgery patients [12,13]. As such, the implementation of effective prophylaxis against DVT assumes paramount importance in mitigating the risk of VTE complications. A multitude of guidelines and recommendations have been established to assist clinicians in selecting the most appropriate prophylactic approach, accounting for factors such as individual patient risk, the nature of the surgical procedure, and other pertinent clinical considerations [14]. Among these recommendations, the American College of Chest Physicians advises the use of mechanical prophylaxis methods, including graduated compression stockings (GCS), intermittent pneumatic compression (IPC), or a combination of mechanical and pharmacological approaches for patients at high risk of DVT [15]. These recommendations are grounded in a comprehensive understanding of the risk factors associated with orthopedic surgery and aim to optimize patient outcomes by reducing the incidence of DVT and its associated complications.

This review is organized into four distinct sections. It begins with an examination of the multifaceted risk factors contributing to the development of DVT in orthopedic patients. Subsequently, it emphasizes the paramount importance of DVT prophylaxis in orthopedic settings, highlighting its clinical significance and direct connection to the previously discussed risk factors. The third section critically assesses the efficacy and safety of various prophylactic methods, encompassing both mechanical and pharmacological approaches. Next, the paper explores the economic dimensions of DVT prophylaxis, including scrutiny of cost-effectiveness and the exploration of strategies for optimization.

## Review

Risk factors for DVT in orthopedic surgery patients

Orthopedic surgery patients face an increased risk of DVT when compared to individuals undergoing other surgical procedures. This increased vulnerability extends to the potential development of PE, chronic venous insufficiency, and post-thrombotic syndromes [16]. Several factors contribute to this increased risk in orthopedic patients, encompassing both surgery-related and patient-related elements [17]. Among surgery-related factors, the duration of the surgical procedure and the extent of immobility experienced by patients in the postoperative period have been identified as significant contributors to DVT risk in the orthopedic context [18]. On the other hand, patient-related factors, such as advanced age, obesity, and a history of prior DVT or PE, represent additional risk factors [11,18,19]. Obesity, for instance, increases the risk of DVT due to its impact on venous return and the elevation of intra-abdominal pressure [20]. Individuals with a history of VTE are at an increased risk of recurrent events following orthopedic surgery, underscoring the importance of proactive risk assessment and prophylaxis.

Orthopedic procedures such as total hip arthroplasty, total knee arthroplasty, and hip fracture surgery are notably associated with an elevated DVT risk when compared to minor orthopedic surgeries, primarily due to their extended operative times, increased tissue trauma, and prolonged postoperative immobility [14,21-23]. Genetic factors also play a role, with conditions like thrombophilia, factor V Leiden, and prothrombin gene mutation elevating the propensity for blood clot formation [24-26]. Beyond genetics, medical conditions such as the use of hormonal therapy, malignancy, and inflammatory disorders such as rheumatoid arthritis contribute to DVT risk by increasing blood viscosity and coagulation tendencies [2,27,28]. For cancer patients undergoing orthopedic procedures, additional cancer-related factors, including chemotherapy, hormone therapy, and metastasis, intensify the susceptibility to DVT [29]. Patients presenting any of these risk factors require vigilant assessment and may necessitate more aggressive DVT prophylaxis to mitigate their heightened risk. Identifying and addressing these factors preoperatively is essential in the prevention of DVT in orthopedic surgery patients.

The importance of DVT prophylaxis in orthopedic surgery patients

Preventing DVT is essential for most orthopedic surgery patients [18]. There are many forms of DVT prophylaxis. Upholding the gold standard in DVT prophylaxis as defined by the American College of Chest Physicians (ACCP) [30], current recommendations underscore a multifaceted approach to mitigating DVT risk in these patients [15,31,32]. The recommended prophylactic methods encompass mechanical approaches involving graduated compression stockings or intermittent pneumatic compression and/or pharmacological prophylaxis with low molecular weight heparin (LMWH) or direct oral anticoagulants (DOACs) [15,31]. Patient risk stratification informs the choice of prophylactic approach, with high-risk individuals benefiting from combined mechanical and pharmacological prophylaxis.

Mechanical prophylaxis involves external devices like graduated compression stockings and intermittent pneumatic compression to promote venous blood flow and prevent stasis in the lower extremities. Graduated compression stockings and intermittent pneumatic compression have shown efficacy in DVT prevention [33] and are generally safe and well-tolerated, with few complications [14]. However, graduated compression stockings may not suit patients with peripheral arterial disease or severe edema, while intermittent pneumatic compression could be contraindicated in certain medical conditions, such as severe congestive heart failure or acute deep-vein thrombosis [14]. Mechanical prophylaxis can be used alone or in combination with pharmacological methods, depending on individual patient risk factors.

Pharmacological prophylaxis involves using anticoagulant medications to prevent blood clot formation through chemical mechanisms. Common options include unfractionated heparin (UFH), LMWH, fondaparinux, and DOACs [32,34]. LMWH is preferred in orthopedic surgery due to its lower bleeding risk after procedures like total hip arthroplasty or total knee arthroplasty [35]. DOACs, a newer anticoagulant class including rivaroxaban, apixaban, edoxaban, and dabigatran, are increasingly used for DVT prophylaxis in orthopedic surgery patients, targeting specific coagulation factors [14]. DOACs have shown non-inferiority to LMWH in both efficacy and safety for total hip arthroplasty and total knee arthroplasty patients [14]. Recent evidence supports LMWH's efficacy, reducing DVT incidence by 60% in hip fracture surgery patients [36]. Rivaroxaban has also demonstrated non-inferiority to LMWH in reducing DVT incidence after total hip arthroplasty [37], and the combination of graduated compression stockings with intermittent pneumatic compression effectively reduces DVT incidence in total knee arthroplasty patients [38].

Combined mechanical and pharmacological prophylaxis involves the simultaneous use of mechanical prophylaxis methods and anticoagulant medications [35]. This approach is typically reserved for patients with multiple risk factors for DVT [39]. The most employed combined prophylaxis methods include the use of graduated compression stockings in conjunction with LMWH or fondaparinux [14]. Randomized trials support the efficacy of combination prophylaxis, particularly strategies involving LMWH, in reducing VTE incidence after gynecologic surgery [40]. Additionally, combining graduated compression stockings with fondaparinux has been associated with a significant reduction in DVT incidence among patients undergoing total hip arthroplasty and total knee arthroplasty [41]. The combined prophylaxis may elevate the risk of bleeding [39], and therefore, the timing of prophylaxis initiation should be guided by individual patient risk factors, medical history, and adherence to evidence-based guidelines. It is imperative to assess the safety, tolerability, and potential side effects associated with each prophylactic measure before administering them. 

Efficacy and safety of DVT prophylaxis in orthopedic surgery patients 

Mechanical prophylaxis measures have demonstrated effectiveness in preventing DVT in patients where pharmacological prophylaxis is contraindicated [42,43]. However, the efficacy of mechanical prophylaxis can be influenced by patient-specific factors such as age, body mass index, and comorbidities [44]. A study observed that the effectiveness of intermittent pneumatic compression in preventing DVT was lower in patients with comorbidities like chronic obstructive pulmonary disease and congestive heart failure [33]. The duration of mechanical prophylaxis remains a topic of debate, but a systematic review and meta-analysis indicated that extended prophylaxis beyond hospital discharge significantly reduced DVT incidence in patients undergoing total hip arthroplasty and total knee arthroplasty [39]. Mechanical compression devices can pose risks, including local soft tissue injury, bleeding, and patient non-compliance [45]. Special attention should be given to the risk of skin complications, which may be heightened in patients with fragile skin or peripheral neuropathy, potentially leading to skin necrosis, falls, compartment syndrome, and peroneal nerve palsy [46-49].

Pharmacological prophylaxis is a widely adopted approach for DVT prevention in orthopedic surgery patients. The effectiveness of LMWH in reducing DVT incidence following hip fracture surgery is well established [50]. Similarly, DOACs are non-inferior to LMWH in both efficacy and safety for patients undergoing total hip arthroplasty or total knee arthroplasty [14]. A systematic review and meta-analysis have shown that fondaparinux significantly reduces DVT incidence in total hip arthroplasty and total knee arthroplasty patients [41]. Combined prophylaxis carries a higher risk of bleeding compared to using prophylactic methods alone [51]. Fondaparinux may be associated with an increased bleeding risk compared to LMWH [52], and heparin use has been linked to bleeding and heparin-induced thrombocytopenia [45]. Several investigations have explored the efficacy of combined mechanical and pharmacological prophylaxis compared to either method alone, particularly in high-risk populations, with combination prophylaxis showing greater effectiveness [53,54]. For instance, a randomized controlled trial found that combining graduated compression stockings with LMWH was more effective in preventing DVT in total knee arthroplasty patients than using each prophylactic method alone [54].

Adverse outcomes stemming from pharmacological prophylaxis can range from minor to life-threatening. Pharmacological prophylaxis, such as LMWH, carries the risk of bleeding complications. The risk of bleeding is higher in patients with a history of bleeding disorders, renal insufficiency, or concurrent use of other anticoagulant medications [55]. Therefore, ensuring patient safety is a primary concern in selecting DVT prophylactic measures for orthopedic patients, and the choice of prophylactic method should be driven by a thorough assessment of the individual patient's risk factors for both DVT and bleeding.

Cost-effectiveness and implementation challenges in DVT prophylaxis

Cost-effectiveness is a primary concern for healthcare providers when choosing a DVT prophylaxis. A study by Schousboe and Brown investigating prophylactic medication options for patients undergoing total hip or knee arthroplasty found aspirin to be a highly effective and cost-efficient choice for thrombosis prevention [56]. Compared to LMWH, aspirin demonstrated better cost-effectiveness while also boasting a superior bleeding profile. However, the selection of prophylactic medication involves considerations beyond economics, and patient-specific factors must weigh into the decision-making process. The American College of Clinical Pharmacy (ACCP) guidelines suggest alternative methods, like unfractionated heparin (UFH), for orthopedic surgery patients with additional DVT risk factors [32]. Implementing proper DVT prophylaxis methods in clinical practice faces various barriers. Various studies underscore the importance of adherence to mechanical and pharmacological protocols, especially when coupled with accurate patient risk assessment for DVT [57]. In one study, venous thrombosis incidence was low with prophylaxis, with a single case occurring in a patient who did not receive pharmacological prophylaxis [57]. To enhance the effectiveness of DVT prophylaxis, risk stratification is pivotal. As recommended by the American College of Chest Physicians (ACCP), a risk stratification model adopts a tiered approach to assess patients' overall thrombotic event risk [58].

Several studies have scrutinized the cost-effectiveness of various DVT prophylactic measures. For instance, Kapoor et al. conducted a study investigating cost differences among multiple DVT prophylaxis modes for patients undergoing total hip or knee arthroplasty [59]. Their findings indicate that extended duration LMWH following total hip replacement and fondaparinux following both total hip replacement and total knee replacement stand out as cost-effective prophylactic regimens. This cost-effectiveness is particularly notable when compared to aspirin and newer oral anticoagulants. Combining aspirin with mechanical compression devices emerges as another cost-effective strategy for DVT prevention. Notably, their combined use following limited tourniquet total knee arthroplasty demonstrated an incidence rate of asymptomatic DVTs reaching as low as 0% [60].

DVT prophylaxis not only curbs the costs linked to DVT treatment but also diminishes the risk of enduring complications. Despite available methods to bolster adherence to DVT prophylaxis protocols, adherence rates remain inconsistent. One institution introduced a computer alert program designed to prompt physicians to prescribe appropriate DVT prophylaxis. This initiative led to increased adherence to relevant policies and a significant reduction in DVT and pulmonary embolism (PE) rates among hospitalized patients at risk [61].

Educating patients about the risks, warning signs, and available treatment methods for DVT prophylaxis represents a cost-effective approach to reducing DVT and PE rates. Research supports this approach, with one study revealing that a targeted, patient-centered education bundle was associated with a decreased rate of non-administration of prophylactic DVT medications [62]. However, it is vital to recognize that patient perceptions of their treatment for DVT prevention play a crucial role. Some studies suggest that patient education, if inadequate, may potentially hinder patient involvement and adherence to DVT prophylaxis protocols. Therefore, healthcare providers must engage in open discussions with patients, ensuring their comprehension of prophylactic treatments and assessing their willingness to participate and comply with specific measures.

## Conclusions

DVT prevention following orthopedic surgery is an integral part of patient care, especially given the fact that orthopedic patients often have limited mobility and are at an increased risk for developing DVT. In addition to this, many patient-specific risk factors may increase an individual's odds of experiencing a DVT following orthopedics surgery. Several methods have been developed to prevent DVT, although not all are equally effective or cost-effective. Pharmacological measures, such as DOACs, LMWH, and mechanical methods of DVT prophylaxis, have demonstrated their efficacy in reducing the risk of DVT among orthopedic surgery patients. Additionally, many patients with multiple risk factors may experience an even more significant benefit from using combined mechanical and pharmacological methods for DVT prophylaxis. It is essential to recognize that the patient's understanding and willingness to adhere to a proposed prophylactic measure is an important step in management. Future studies are needed to assess the efficacy of newer pharmacological and mechanical approaches in preventing DVT and reducing the risk of PE. More studies are needed to explore the long-term outcomes of orthopedic surgery patients to help determine the optimal prophylaxis. A patient-centered approach, integrating tailored patient education, selecting cost-effective medications personalized for individual patients, and widespread utilization, holds promise for further reducing DVT rates among orthopedic patients in the future.

## References

[1] (2023). Data and statistics on venous thromboembolism. https://www.cdc.gov/ncbddd/dvt/data.html.

[2] Kahn SR, Lim W, Dunn AS (2012). Prevention of VTE in nonsurgical patients: antithrombotic therapy and prevention of thrombosis, 9th Ed: American College of Chest Physicians evidence-based clinical practice guidelines. Chest.

[3] Haas SB, Barrack RL, Westrich G (2009). Venous thromboembolic disease after total hip and knee arthroplasty. Instr Course Lect.

[4] Lowe GD (2003). Virchow's triad revisited: abnormal flow. Pathophysiol Haemost Thromb.

[5] Kuipers S, Cannegieter SC, Middeldorp S, Robyn L, Büller HR, Rosendaal FR (2007). The absolute risk of venous thrombosis after air travel: a cohort study of 8,755 employees of international organisations. PLoS Med.

[6] McLendon K, Goyal A, Attia M (2023). Deep Venous Thrombosis Risk Factors. StatPearls.

[7] Stone J, Hangge P, Albadawi H (2017). Deep vein thrombosis: pathogenesis, diagnosis, and medical management. Cardiovasc Diagn Ther.

[8] Kernan WN, Ovbiagele B, Black HR (2014). Guidelines for the prevention of stroke in patients with stroke and transient ischemic attack: a guideline for healthcare professionals from the American Heart Association/American Stroke Association. Stroke.

[9] Warwick D, Rosencher N (2010). The ''critical thrombosis period'' in major orthopedic surgery: when to start and when to stop prophylaxis. Clin Appl Thromb Hemost.

[10] Lee SY, Ro du H, Chung CY, Lee KM, Kwon SS, Sung KH, Park MS (2015). Incidence of deep vein thrombosis after major lower limb orthopedic surgery: analysis of a nationwide claim registry. Yonsei Med J.

[11] Motohashi M, Adachi A, Takigami K, Yasuda K, Inoue M, Sasaki S, Matsui Y (2012). Deep vein thrombosis in orthopedic surgery of the lower extremities. Ann Vasc Dis.

[12] Heit JA, Silverstein MD, Mohr DN, Petterson TM, O'Fallon WM, Melton LJ 3rd (1999). Predictors of survival after deep vein thrombosis and pulmonary embolism: a population-based, cohort study. Arch Intern Med.

[13] Saleh J, El-Othmani MM, Saleh KJ (2017). Deep vein thrombosis and pulmonary embolism considerations in orthopedic surgery. Orthop Clin North Am.

[14] Kim HJ, Walcott-Sapp S, Leggett K, Bass A, Adler RS, Pavlov H, Westrich GH (2010). Detection of Pulmonary Embolism in the Postoperative Orthopedic Patient Using Spiral CT Scans. HSS J.

[15] Falck-Ytter Y, Francis CW, Johanson NA (2012). Prevention of VTE in orthopedic surgery patients: Antithrombotic Therapy and Prevention of Thrombosis, 9th ed: American College of Chest Physicians Evidence-Based Clinical Practice Guidelines. Chest.

[16] Guyatt GH, Akl EA, Crowther M, Gutterman DD, Schuünemann HJ (2012). Executive summary: Antithrombotic Therapy and Prevention of Thrombosis, 9th ed: American College of Chest Physicians Evidence-Based Clinical Practice Guidelines. Chest.

[17] Geerts WH, Pineo GF, Heit JA, Bergqvist D, Lassen MR, Colwell CW, Ray JG (2004). Prevention of venous thromboembolism: the Seventh ACCP Conference on Antithrombotic and Thrombolytic Therapy. Chest.

[18] Anderson DR, Morgano GP, Bennett C (2019). American Society of Hematology 2019 guidelines for management of venous thromboembolism: prevention of venous thromboembolism in surgical hospitalized patients. Blood Adv.

[19] Bui MH, Hung DD, Vinh PQ, Hiep NH, Anh LL, Dinh TC (2019). Frequency and Risk Factor of Lower-limb Deep Vein Thrombosis after Major Orthopedic Surgery in Vietnamese Patients. Open Access Maced J Med Sci.

[20] Jacobs JJ, Mont MA, Bozic KJ (2012). American Academy of Orthopaedic Surgeons clinical practice guideline on: preventing venous thromboembolic disease in patients undergoing elective hip and knee arthroplasty. J Bone Joint Surg Am.

[21] Wells PS, Anderson DR, Rodger M (2000). Derivation of a simple clinical model to categorize patients probability of pulmonary embolism: increasing the models utility with the SimpliRED D-dimer. Thromb Haemost.

[22] Zhang ZH, Shen B, Yang J, Zhou ZK, Kang PD, Pei FX (2015). Risk factors for venous thromboembolism of total hip arthroplasty and total knee arthroplasty: a systematic review of evidences in ten years. BMC Musculoskelet Disord.

[23] Greenfield LJ, Wakefield TW (1989). Prevention of venous thrombosis and pulmonary embolism. Adv Surg.

[24] Cohen AT (2010). Asia-Pacific Thrombosis Advisory Board consensus paper on prevention of venous thromboembolism after major orthopaedic surgery. Thromb Haemost.

[25] Whiting PS, White-Dzuro GA, Greenberg SE, VanHouten JP, Avilucea FR, Obremskey WT, Sethi MK (2016). Risk factors for deep venous thrombosis following orthopaedic trauma surgery: an analysis of 56,000 patients. Arch Trauma Res.

[26] Yu X, Wu Y, Ning R (2021). The deep vein thrombosis of lower limb after total hip arthroplasty: what should we care. BMC Musculoskelet Disord.

[27] White RH, Romano PS, Zhou H, Rodrigo J, Bargar W (1998). Incidence and time course of thromboembolic outcomes following total hip or knee arthroplasty. Arch Intern Med.

[28] Rahman A, Islam AM, Husnayen S (2012). Recurrent deep vein thrombosis due to thrombophilia. Korean Circ J.

[29] Khan M, Altaf C, Saeed Malik H, Abdul Naeem M, Latif A (2021). Heritable thrombophilia in venous thromboembolism in northern Pakistan: a cross-sectional study. Adv Hematol.

[30] Mahé I, Chidiac J, Bertoletti L (2017). The clinical course of venous thromboembolism may differ according to cancer site. Am J Med.

[31] Abdol Razak NB, Jones G, Bhandari M, Berndt MC, Metharom P (2018). Cancer-associated thrombosis: An overview of mechanisms, risk factors, and treatment. Cancers (Basel).

[32] LaVasseur C, Neukam S, Kartika T, Samuelson Bannow B, Shatzel J, DeLoughery TG (2022). Hormonal therapies and venous thrombosis: considerations for prevention and management. Res Pract Thromb Haemost.

[33] Lyman GH, Khorana AA, Kuderer NM (2013). Venous thromboembolism prophylaxis and treatment in patients with cancer: American Society of Clinical Oncology clinical practice guideline update. J Clin Oncol.

[34] Kearon C, Akl EA, Comerota AJ (2012). Antithrombotic therapy for VTE disease: Antithrombotic Therapy and Prevention of Thrombosis, 9th ed: American College of Chest Physicians Evidence-Based Clinical Practice Guidelines. Chest.

[35] Kahn SR, Shivakumar S (2020). What's new in VTE risk and prevention in orthopedic surgery. Res Pract Thromb Haemost.

[36] Flevas DA, Megaloikonomos PD, Dimopoulos L, Mitsiokapa E, Koulouvaris P, Mavrogenis AF (2018). Thromboembolism prophylaxis in orthopaedics: an update. EFORT Open Rev.

[37] Gould MK, Garcia DA, Wren SM, Karanicolas PJ, Arcelus JI, Heit JA, Samama CM (2012). Prevention of VTE in nonorthopedic surgical patients: antithrombotic therapy and prevention of thrombosis, 9th Ed: American College of Chest Physicians evidence-based clinical practice guidelines. Chest.

[38] Kamphuisen PW, Agnelli G (2007). What is the optimal pharmacological prophylaxis for the prevention of deep-vein thrombosis and pulmonary embolism in patients with acute ischemic stroke?. Thromb Res.

[39] Lu X, Lin J (2018). Low molecular weight heparin versus other anti-thrombotic agents for prevention of venous thromboembolic events after total hip or total knee replacement surgery: a systematic review and meta-analysis. BMC Musculoskelet Disord.

[40] Mismetti P, Laporte S, Darmon JY, Buchmüller A, Decousus H (2001). Meta-analysis of low molecular weight heparin in the prevention of venous thromboembolism in general surgery. Br J Surg.

[41] Sang CQ, Zhao N, Zhang J (2018). Different combination strategies for prophylaxis of venous thromboembolism in patients: a prospective multicenter randomized controlled study. Sci Rep.

[42] Hull RD, Pineo GF, Stein PD (2001). Extended out-of-hospital low-molecular-weight heparin prophylaxis against deep venous thrombosis in patients after elective hip arthroplasty: a systematic review. Ann Intern Med.

[43] Caprini JA (2010). Mechanical methods for thrombosis prophylaxis. Clin Appl Thromb Hemost.

[44] Nicholson M, Chan N, Bhagirath V, Ginsberg J (2020). Prevention of venous thromboembolism in 2020 and beyond. J Clin Med.

[45] Ganau M, Ligarotti GK, Meloni M, Chibbaro S (2019). Efficacy and safety profiles of mechanical and pharmacological thromboprophylaxis. Ann Transl Med.

[46] Datta I, Ball CG, Rudmik L, Hameed SM, Kortbeek JB (2010). Complications related to deep venous thrombosis prophylaxis in trauma: a systematic review of the literature. J Trauma Manag Outcomes.

[47] Weinberger J, Cipolle M (2016). Mechanical prophylaxis for post-traumatic VTE: stockings and pumps. Curr Trauma Rep.

[48] Siddiqui AU, Buchman TG, Hotchkiss RS (2000). Pulmonary embolism as a consequence of applying sequential compression device on legs in a patient asymptomatic of deep vein thrombosis. Anesthesiology.

[49] Ringley CD, Johanning JM, Gruenberg JC, Veverka TJ, Barber KR (2002). Evaluation of pulmonary arterial catheter parameters utilizing intermittent pneumatic compression boots in congestive heart failure. Am Surg.

[50] Boelig MM, Streiff MB, Hobson DB, Kraus PS, Pronovost PJ, Haut ER (2011). Are sequential compression devices commonly associated with in-hospital falls? A myth-busters review using the patient safety net database. J Patient Saf.

[51] Handoll HH, Farrar MJ, McBirnie J, Tytherleigh-Strong GM, Milne AA, Gillespie WJ (2002). Heparin, low molecular weight heparin and physical methods for preventing deep vein thrombosis and pulmonary embolism following surgery for hip fractures. Cochrane Database Syst Rev.

[52] Perrotta C, Chahla J, Badariotti G, Ramos J (2020). Interventions for preventing venous thromboembolism in adults undergoing knee arthroscopy. Cochrane Database Syst Rev.

[53] Kumar A, Talwar A, Farley JF, Muzumdar J, Schommer JC, Balkrishnan R, Wu W (2019). Fondaparinux sodium compared with low-molecular-weight heparins for perioperative surgical thromboprophylaxis: a systematic review and meta-analysis. J Am Heart Assoc.

[54] Davila JG, Mitchell WB, Morrone KA (2021). Venous thromboembolism prophylaxis practices for patients with sickle cell disease pre and during the COVID-19 pandemic. Blood.

[55] Kakkos S, Kirkilesis G, Caprini JA, Geroulakos G, Nicolaides A, Stansby G, Reddy DJ (2022). Combined intermittent pneumatic leg compression and pharmacological prophylaxis for prevention of venous thromboembolism. Cochrane Database Syst Rev.

[56] Joubert F, Gillois P, Bouaziz H, Marret E, Iohom G, Albaladejo P (2019). Bleeding complications following peripheral regional anaesthesia in patients treated with anticoagulants or antiplatelet agents: a systematic review. Anaesth Crit Care Pain Med.

[57] Schousboe JT, Brown GA (2013). Cost-effectiveness of low-molecular-weight heparin compared with aspirin for prophylaxis against venous thromboembolism after total joint arthroplasty. J Bone Joint Surg Am.

[58] Akhtar AB, Mehdi SR, Khan A, Zahid MT, Abu Bakar M (2021). Clinical practice for venous thromboembolism prophylaxis in patients undergoing oncological surgeries. Cureus.

[59] Laryea J, Champagne B (2013). Venous thromboembolism prophylaxis. Clin Colon Rectal Surg.

[60] Kapoor A, Chuang W, Radhakrishnan N (2010). Cost effectiveness of venous thromboembolism pharmacological prophylaxis in total hip and knee replacement: a systematic review. Pharmacoeconomics.

[61] Snyder MA, Sympson AN, Scheuerman CM, Gregg JL, Hussain LR (2017). Efficacy in deep vein thrombosis prevention with extended mechanical compression device therapy and prophylactic aspirin following total knee arthroplasty: a randomized control trial. J Arthroplasty.

[62] Kucher N, Koo S, Quiroz R, Cooper JM, Paterno MD, Soukonnikov B, Goldhaber SZ (2005). Electronic alerts to prevent venous thromboembolism among hospitalized patients. N Engl J Med.

